# Medical Therapy of Heart Failure with Reduced Ejection Fraction—A Call for Comparative Research

**DOI:** 10.3390/jcm10091803

**Published:** 2021-04-21

**Authors:** Gad Cotter, Beth A. Davison, Alexandre Mebazaa, Koji Takagi, Maria Novosadova, Yonathan Freund, Alain Cohen-Solal

**Affiliations:** 1 Momentum Research, Inc., 50101 Governors Drive, Chapel Hill, NC 27517, USA; BethDavison@momentum-research.com (B.A.D.); kojitakagi@momentum-research.com (K.T.); marianovosadova@momentum-research.com (M.N.); 2 Inserm U942-MASCOT, 75010 Paris, France; alexandre.Mebazaa@aphp.fr; 3 Department of Anesthesiology and Critical Care Medicine, AP-HP, Saint Louis Lariboisière University Hospitals, 75010 Paris, France; 4 School of Medicine, Université de Paris, 75010 Paris, France; 5 School of Medicine, Sorbonne University, 75005 Paris, France; yonathan.freund@aphp.fr; 6 Emergency Department, Hopital Pitié-Salpêtrière, 75013 Paris, France; 7 Service de Cardiologie, Hôpital Lariboisière, 2 Rue Ambroise Paré, 75010 Paris, France; alain.cohen-solal@aphp.fr

**Keywords:** heart failure, heart failure reduced ejection fraction, treatment, blockers of rennin angiotensin system, beta blockers

## Abstract

The armamentarium of therapies for patients with heart failure and reduced ejection fraction (HFREF) has increase substantially with the introduction of Angiotensin Receptor Neprilysin Inhibitor (ARNi), sodium glucose cotransport inhibitors (SGLTis), ivabradine, and Vericinguat, bringing to seven the number of potential therapies for HFREF. In the current review we highlight available data on the different classes of medications. Renin angiotensin blockers (RAASbs) and beta blockers (BBs) were shown to have very substantial effects in patients with HFREF. These medications are generic and hence relatively inexpensive. They have a 30-year track record of relatively benign short- and long-term safety profiles and should remain the cornerstone of therapy for patients with HFREF. ARNis are effective in further reducing adverse effects and should replace RAASbs in symptomatic HFREF patients, despite their relatively high prices. The addition of SGLTis (congested patients), Ivabradine (tachycardic patients), and Vericinguat (hypertensive patients) should be considered in patients who remain symptomatic despite optimal doses of RAASbs/ARNis, MRAs, and BBs. Comparative studies examining the efficacy of these medications, and strategies and prioritizing some over others should be considered as, given their similar side effects on heart rate, blood pressure, and renal function, it is highly unlikely that all can be given to the same patient.

## 1. Introduction

With the publication of the SOLVD study [1] in 1991, the treatment of heart failure and reduced ejection fraction (HFREF) shifted from symptomatic relief (with diuretics) to disease-modifying therapies aimed at improving outcomes. The outcomes perceived as most important to modify in HFREF are death, especially cardiovascular death, and heart failure (HF) admissions. After the SOLVD study [1], other modifiers of the renin angiotensin system (RAASbs) were introduced, followed by beta blockers (BBs) and finally mineralocorticoids receptor antagonists (MRAs, [2]). In the last 10 years, many new medications have been recommended for patients with HFREF. First, ivabradine was shown to reduce HF admission (but not mortality) in patients with HFREF [3]. Then, in 2014, the PARADIGM study [4] was published in which patients with HFREF who were routinely treated with “triple therapy” including RAASb, BB, and MRA were randomized to the Angiotensin Receptor Neprilysin Inhibitor (ARNi) sacubitril/valsartan or enalapril. The study results showed that patients treated with sacubitril/valsartan had a further reduction (as compared to the RAASb enalapril) in HF readmissions and deaths. In the last 2 years, at least three additional medications were shown to be effective and then approved. First, sodium glucose cotransport inhibitors (SGLTis) were shown to induce a large reduction in HF readmissions and some reductions in deaths [5,6], then vericiguat was shown to reduce a combined endpoint of HF readmission or death [7], and finally ferric carboxymaltose was shown to reduce heart failure readmissions after an AHF admission in patients with iron deficiency and a recent acute HF [8] admission. Given this plethora of available medications, which ones should be administered to patients with HFREF and in what order?

## 2. Safety and Effect Size

The first consideration when building a therapy for a patient with HFREF is the benefit to risk ratio of the available therapies, i.e., what do we know about the effect sizes of the available medications and what do we know about their safety? As stated above, RAASbs have been used in the treatment of patients with HFREF for over 30 years [2]. Their safety profile is very well known, and the up-titration strategy is familiar to all physicians treating such patients. The results of the SOLVD study showed that RAASbs are highly effective. The number needed to treat (NNT) with a RAASb to prevent an adverse event of HF admission or death was found to be around 6, i.e., for every six patients treated for one year, one HF admission or death will be prevented each year. This NNT is one of the most favorable in clinical medicine. This very favorable risk benefit ratio with a substantial benefit and largely benign risk profile strongly puts these medications as first-line interventions in patients with HFREF. Some have argued that safety concerns have limited the use of RAASbs. However, these concerns are largely unsubstantiated. In the SOLVD study, for instance [1], systolic blood pressure decreased by 4 mmHg on average, and less than 3% of patients developed significant hyperkalemia or renal impairment [1]. Hence, RAASbs are highly effective and safe. The same can be said regarding BBs. Studies done in the 1990s have shown similar efficacy to that of RAASbs. Although BBs have some more troublesome side effects (such as bradycardia, worsening of HF, hypotension) when initiated, a careful up-titration can usually allow their administration in most patients with HFREF. Importantly, the NNT for BBs when added to RAASbs was also extremely low, at six per year. The efficacy of MRAs was examined in patients with HFREF already treated by RAASbs and BBs. The additional benefits were found to be modest, with an NNT of around 20. The side effects of MRAs are also manageable, although some have argued that in “real life”, the side effects of hyperkalemia are quite common and the introduction of these medications should be done with extreme care [9].

In 2010, the SHIFT study [3] was published, showing that in patients with HFREF and a heart rate of >70 beats per minute, the addition of ivabradine resulted in slowing of the heart rate and reduction of HF admissions but not mortality. In 2014, the results of the PARADIGM-HF study were published [4]. In this study, the ARNi sacubitril/valsartan was compared with enalapril in patients with HFREF. The results showed a further reduction of 20% in both HF admissions and deaths in patients treated with sacubitril/valsartan. The NNT was modest, around 20; however, this improvement was achieved on top of that of a RAASb as the control arm received good doses of enalapril. This has led to the suggestion [2] that sacubitril/valsartan should replace older RAASbs as the first line therapy of patients with HFREF who are symptomatic despite treatment with BBs, RAASbs, and MRAs. In the last 2 years, two studies have been published demonstrating that, in patients with HFREF already treated by RAASb/ARNi + BB + MRA, the addition of SGLTis leads to further improvements in outcomes, most importantly quite substantial reductions in HF admissions and reductions in mortality [5,6]. These findings have led to the approval of SGLTis for the treatment of patients with HFREF as a second-line therapy. Some experts have lauded the extreme safety of SGLTis. However, examining the data from the SGLTis HF studies, one notes that, although less pronounced then RAASbs, patients initiated on SGLTis had some drops in BP and creatine increases during the first year after initiation. Finally, recently, the Victoria study has been published [7], examining the effects of bericinguat in patients with HFREF and a recent HF exacerbation. The study has shown some modest efficacy, with a NNT of about 25 and no substantial side effects beyond some hypotension. Again, the effects of vericiguat were examined on top of therapy with ACEI, BBS, and MRAs.

Given the above study designs, in which newer therapies were initiated in stable patients already treated with all available medications at the time, it is assumed that a stepwise approach will be utilized in which patients will be first initiated on “triple therapy” with RAASb/ARNi + BB + MRA. In addition, in those with a heart rate of >70 despite optimization of BBs or who cannot tolerate full doses of BBs, ivabradine may be considered. SGLTis would be added to symptomatic patients who can tolerate them when treated with the above-mentioned medications, and vericiguat may be considered for further intensification in symptomatic patients, especially those who are stable and have preserved blood pressure. However, it has recently been suggested that SGLTis should be made a first-line therapy in patients with HFREF [10]. The question is, should this be considered? First, the safety data available for SGLTis in patients with HFREF are limited, as the drugs are novel, only used in patients already treated with triple therapy of RAASb/ARNi + BB + MRA. Although the HF studies of SGLTis [5,6] suggest a relatively benign safety profile with a small number of patients developing hypotension, small reductions in eGFR in the first year of therapy (followed by improvement), and no hypokalemia, some infections were observed. Second, the effect sizes in patients already treated with triple therapy were modest for SGLTis—in the ballpark of NNT = 20. As stated above, the NNT observed with RAASbs as a first-line therapy for HFREF was around six. We do not know what the effects would be of reversing the order and replacing RAASbs/ARNis with SGLTis.

## 3. Dose Optimization vs. Initiation of Newer Therapies

Most patients treated for HFREF are treated with suboptimal doses of HF medications [11], although one study [12] suggests that dose optimization does not further reduce mortality but substantially affects HF admissions. Hence, the question of adding new medications, vis-a-vie optimization of existing HF medications, has not been resolved. In this respect, the currently ongoing STRONG-HF study may help address the question [13]. In this study, patients with a recent admission for AHF who are not optimally treated with triple therapy (RAASbs, BBS and MRAS) whatever LVEF, are randomized to usual care versus a strategy of rapid up-titration of these medications under frequent clinical and laboratory assessments including natriuretic peptides. The study will examine quality of life as well as outcomes such as HF admissions and death. In the meantime, we do not know whether higher “optimal “doses of HF medications are superior to the lower doses used currently in day-to-day routine treatment of patients with HFREF.

## 4. Quality of Life

An important consideration in the treatment of HFREF patients is quality of life (QoL). Early research into new treatments for HFREF focused only on improving outcomes (HF admission and deaths), and data on effects on quality of life has been scarce. We believe, based on existing limited data, that RAASbs [14] improve quality of life in patients with HFREF through reducing some bothersome symptoms. On the other hand, we believe that the same cannot be said as regards BBs, although the studies assessing the effects of BBs on OqL were small. Equally, little is known of the effects of MRAs on QoL. Some data was reported in later studies such as TOPCAT [15], although TOPCAT was performed in patients with preserved ejection fraction. The information on the effects on QoL is better when it comes to newer studies. The ARNi sacubitril/valsartan [16], ibavaradine [17], and SGLTis [5,6] were all found to have significant beneficial effects on QoL.

Given the fact that patients with HFREF treated with the above-mentioned therapies live longer and have fewer HF admissions, the focus of therapy to some degree has moved in recent years from only preventing adverse outcomes to preventing those outcomes while also improving quality of life. In this respect, we believe that tailoring the available therapies to the specific patient is critical. In many cases, symptomatic patients have been treated up to 10 years ago by increasing doses of diuretics and reduction in the doses of BBs. This avenue should, in our opinion, be reconsidered, given the effects of newer therapies on QoL. First, optimization of RAASbs and MRAs, which probably have beneficial effects on QoL, should be attempted. In parallel, given the significant benefits seen with ivabradine, ARNIs, and SGLTis on symptoms and QoL, these therapies should probably be considered as an alternative or a supplement to higher doses of RAASbs and MRAs in symptomatic patients with HFREF. We believe that patients who have significant symptomatic burden should be considered for early switching of their RAASb therapy to an ARNi and the addition of a SGLTi. In those who have a higher heart rate and where BBs cannot be further up-titrated due to side effects, ivabradine should be considered.

## 5. Recent Worsening of HF

In many cases, patients with HFREF who are not optimally treated are encountered during assessment due to progressive symptoms, either in the outpatient clinic (worsening HF) or as inpatients (during or after an admission for AHF). What do we know of the best approach to such patients?

Importantly, one has to recognize that all the above-mentioned therapies (with small exceptions that will be highlighted below) were developed in patients who were very stable. Indeed, all initial RAASbs, BBs, ivabradine, ARNIs, and SGLTis studies have explicitly excluded patients during or shortly after any HF exacerbation or AHF admission. Hence, the relative efficacy of these therapies in unstable patients is largely not known. Absent results of the ongoing STRONG-HF study [12] examining the effects of higher doses of RAASbs, BBs, and MRAs immediately after an AHF admission, in patients with a recent HF exacerbation, efforts should be made to optimize, as much as possible, therapy with known effective medications for HFREF. With regards to newer therapies, a few options should be considered [18].

First, the ARNi sacubitril/valsartan was assessed in a small study of patients admitted for AHF and was found to be quite effective in reducing natriuretic peptide levels and improving some outcomes [19]. Despite the study being small, and the outcome assessment being limited by their post-hoc nature, we believe that these data should encourage the initiation of sacubitril/valsartan early in patients who have had a recent worsening of HF, especially given the substantial beneficial effects of sacubitril/valsartan on symptoms and QoL. Second, the SGLTi sotaglifozin was found to be extremely effective in patients with a recent AHF admission, with NNT in the vicinity of 5—better than any HF therapy ever developed [20]. However, sotaglifozin was tested only in patients with diabetes melitus and is not currently available for patients with HF (the drug is not marketed as of now). Of note, sotaglifozin is a combined SGLT 1+2 inhibitor, while the other SGLTis available now are only SGLT2 inhibitors. Whether the results with sotaglifozin can be extrapolated to SGLTis with SGLT2-only effects is not clear. A recent analysis from the DAPA HF study [21] has suggested that most of the effect of SGLTs occurs early, during the first months after initiation, especially when it comes to HF events, and a very small study suggested that empafligozin reduces the event rate of worsening HF, HF readmission, and death during the first 30 days after admission in patients with AHF [22]. In addition, given the significant effects of SGLTis on QoL and the fact that these drugs are especially effective in reducing further HF admission, it would seem prudent to introduce these medications early after a HF exacerbation. Third, vericiguat was tested in patients during or after a HF admission. A recent analysis has suggested that the drug is not effective in patients who were initiated during an AHF admission and, hence, vericiguat is probably not a good option for patients with a recent HF exacerbation [23]. Finally, in a recent study it was shown that administration of ferric carboxymaltose to patients after an AHF admission reduces HF readmission, especially in patients with lower baseline ferritin [8].

In summary, we believe that patients who have had a recent HF exacerbation should be optimized as soon as possible regarding all their existing therapies for HFREF inclusive RAASbs, BBs, and MRAS. Early switching of their RAASbs to an ARNi and addition of SGLTis, especially if symptoms persist, should be considered. In those with lower ferritin, ferric carboxymaltose should be administered.

## 6. A Global Approach, Cost Implications and Comparative Research

Newer medications such as ivabradine, ARNis, SGLTis, and vericiguat are expensive and hence not available globally. We believe that over 95% of HFREF patients globally have no access to these therapies. Importantly, even when these medications are available, they have very significant cost implications for health care delivery given the high prices these medications command. Therefore, comparative studies may be needed to assess the true impact of these medications in different settings.

First, if the STRONG-HF study shows that higher doses of traditional RAASbs, MRAs, and BBs are more effective then lower doses, it will be imperative to assess the effects of ARNIs, and especially SGLTis, when added to such higher doses. Second, the question of ordering the initiation of these drugs will become even more important. Given that all medications for HFREF reduce blood pressure, and some also have effects on kidney function and heart rate, one has to consider carefully which should be initiated first as, after 1–2 medications are prescribed, the patients’ tolerance to additional therapies may become compromised and second- or third-line medications may not be tolerated. This is especially true in the setting of a HF exacerbation, where patients who are not optimally treated are often identified.

Therefore, we believe that physicians and health providers caring for patients with HFREF should prioritizes prospective comparative studies. Such studies should primarily be conducted in patients with a recent HF exacerbation and focus on different treatment strategies. Examples of such strategies include comparison of optimized doses of simple, cheap generic triple therapy with older RAASbs, BBs, and MRAs versus upfront initiation of SGLTis, ARNis, and their combinations. Consideration can also be given to the early addition of ivabradine to patients with significant tachycardia or vericiguat to patients with stable HF and higher blood pressure.

Only through comparative prospective randomized studies will we be able to determine which medications—and in which order—should be administered to patients with HF for whom therapeutic options have increased exponentially, close to (although not there yet) as many as the stars in the sky.

## Data Availability

Not applicable.
